# Supplementary material for: Transient Receptor Potential Channel Expression Signatures in Tumor-Derived Endothelial Cells: Functional Roles in Prostate Cancer Angiogenesis
Source: Cancers (Basel). 2019 Jul 8;11(7):956. doi: 10.3390/cancers11070956 (PMC6678088; doi:10.3390/cancers11070956)
Supplement: Supplementary file 1 [file cancers-11-00956-s001.pdf]

Supplementary Materials

Transient Receptor Potential Channel Expression Signatures in Tumor-Derived Endothelial Cells: Functional Roles in Prostate Cancer Angiogenesis

Michela Bernardini, Alessia Brossa, Giorgia Chinigò, Guillaume P. Grolez, Giulia Trimaglio, Laurent Allart, Audrey Hulot, Guillemette Marot, Tullio Genova, Aditi Joshi, Virginie Mattot, Gaelle Fromont, Luca Munaron, Benedetta Bussolati, Natalia Prevarskaya, Alessandra Fiorio Pla and Dimitra Gkika

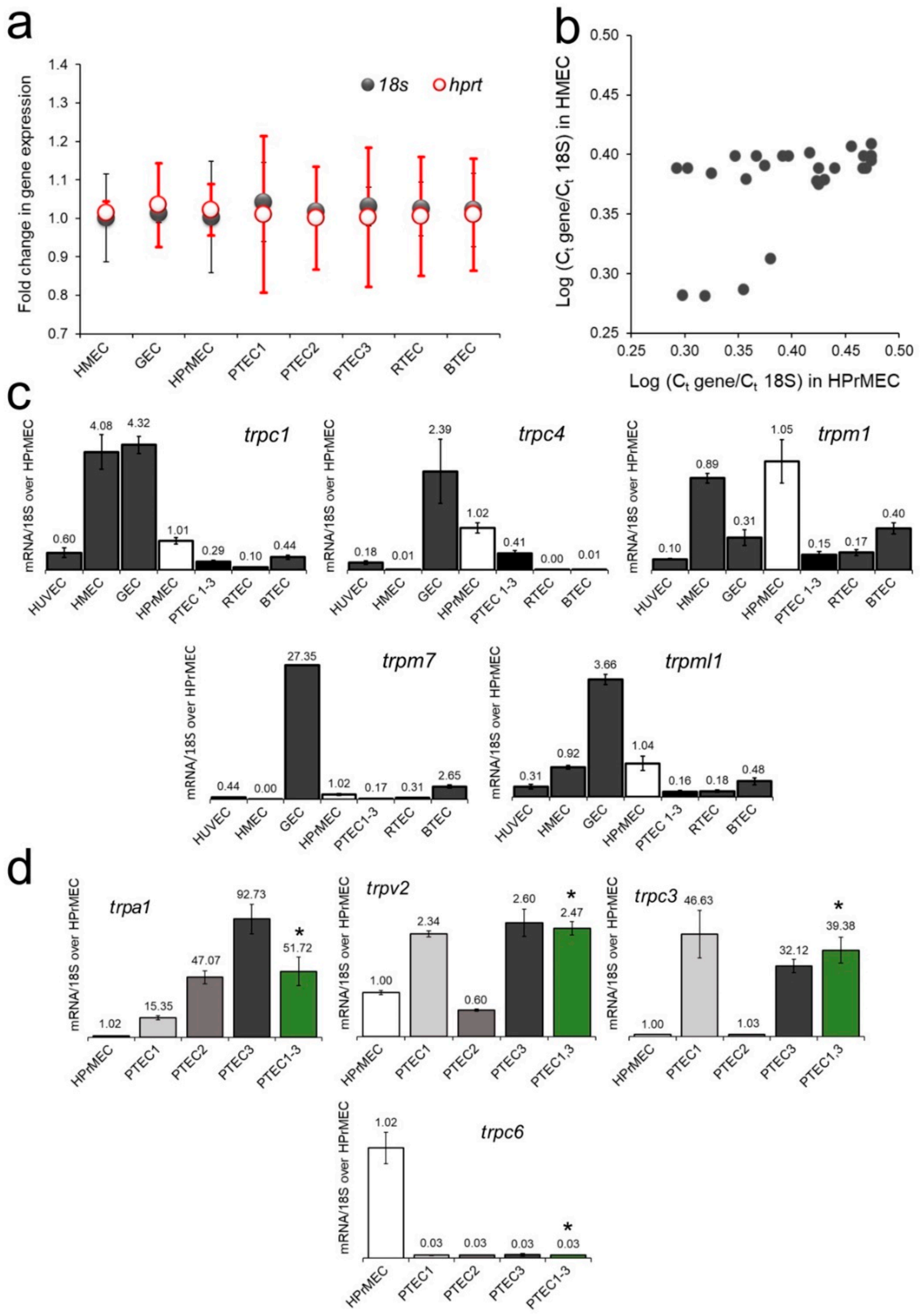

Figure S1. Assessment of external and internal controls of real-time qPCR used for the normalization of expression data. (**a**) Bar plot showing the relative amount of *18s* and *hprt* mRNAs in normal ECs (HMEC, GEC, and HPrMEC), and in TECs (PTEC1, PTEC2, PTEC3, RTECs, and BTEC). Values are

expressed as the mean ΔΔCT values ± SEM of at least three independent experiments. (**b**) Scatter plots showing the mRNA expression profiles of HPrMEC and HMEC for *trp* channels, as determined by real-time qPCR. Correlation values for *trp* channels are expressed as $Log\ (\frac{C_{T,gene\ x}}{C_{T,18s}})$ in normal primary HPrMEC (*x*-axis) and normal HMEC (*y*-axis). (**c**) Real-time qPCR analysis of mRNA expression showing that three *trp* genes (i.e., *trpc1*, *trpc4*, and *trpml1*) are downregulated in all TECs compared to their normal counterparts, while *trpm1* is downregulated in PTEC and BTEC but upregulated in RTEC and *trpm7* is downregulated in PTEC and RTEC but upregulated in BTEC. Values are expressed as the mean ΔΔCT values ± SEM of at least three independent experiments. (**d**) mRNA expression profiles from real-time qPCR analysis normalized against the profile in HPrMEC. Values are expressed as the mean ΔΔCT values±SEM. of at least three independent experiments. Statistical significance *: *p*-value < 0.05.

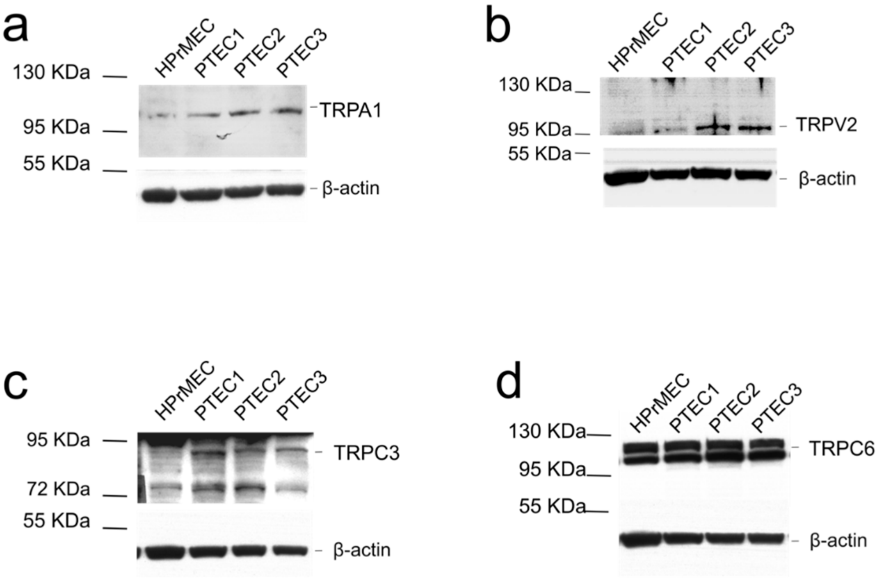

Figure S2. Representative immunoblots blots showing TRPA1 (n = 3) (**a**), TRPV2 (n = 5) (**b**), TRPC3 (n = 3) (**c**), and TRPC6. (n = 3) (**d**) expression in HPrMEC and PTEC1, 2 and 3.

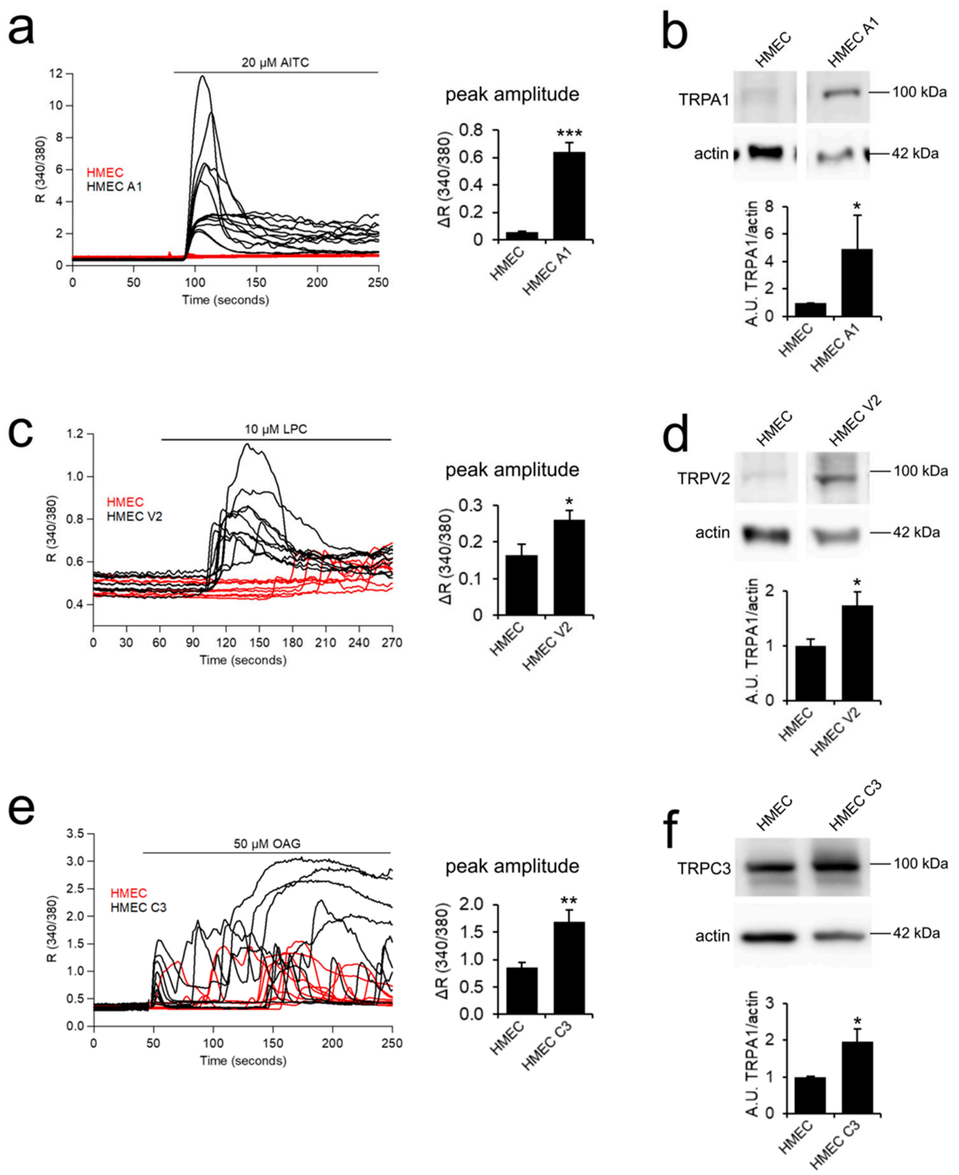

Figure S3. Validation of '*prostate-associated*' channel overexpression in HMECs for their functional characterization in ECs. Each panel shows Ca^{2+} imaging and western blotting results, with relative quantifications, of control HMECs and HMECs overexpressing the '*prostate-associated*' channels. As a control, HMEC were transfected with the empty vector. Ca^{2+} imaging experiments of control HMEC and HMEC-overexpressing the channels show differential channel activity. Peak amplitude was evaluated as the mean value ± SEM recorded after channel activation with 20 µM AITC for TRPA1 (**a**) and 10 µM LPC for TRPV2 (**c**). For TRPC3 (**e**), each trace represents the ratio (340/380 nm) of a single cell in the field in one representative experiment recorded after channel activation with 50 µM OAG based on at least three independent experiments. Calcium imaging was performed 24 and 48 h after transfection. (**b**, **d** and **f**) Western blots show one representative experiment performed 24 h after transfection. Relative quantifications are the mean ± SEM of at least three independent experiments. Statistical significance *: p-value < 0.05, **: p-value < 0.005 and ***: p-value < 0.0005 vs. HMECs.

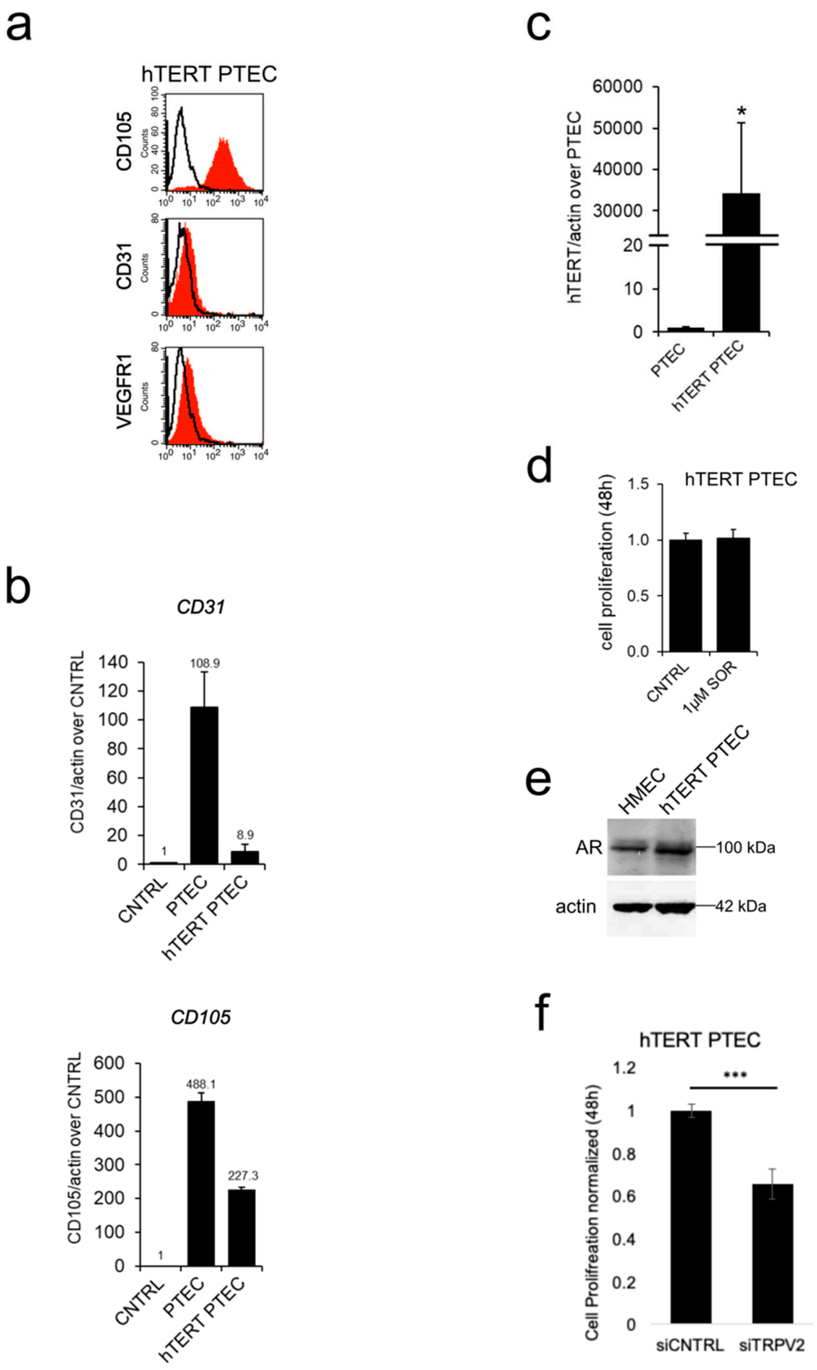

Figure S4. hTERT PTEC characterization. (**a**) Representative cytofluorimetric analysis of the expression of endothelial markers (CD105, CD31, VEGFR1) in hTERT PTECs at passage 7. (**b**) Representative qPCR analysis of the expression of endothelial markers (CD31 and CD105) in control (total renal tumor cells, CNTRL), primary PTEC and hTERT PTEC at passage 7. (**c**) hTERT PTEC show that hTERT mRNA is upregulated compared with that in wild-type PTECas detected by qPCR experiments. (**d**) Cell viability experiments showed that hTERT PTEC are resistant to 1 μM sorafenib 48 h after treatment. Proliferation was detected by MTS assays at 48 h after transfection. Data represented the mean ± SEM of a minimum of three independent experiments. Statistical significance *: p-value < 0.05. (**e**) Validation of *'prostate-associated'* channels and androgen receptor (AR) expression in HMEC and hTERT PTEC. Actin (42 kDa) was used as a loading control. (**f**) Cell proliferation experiments showed that TRPV2 downregulation in hTERT PTEC significantly inhibits cell proliferation. Proliferation was detected by BrDU assays at 48 h after siTRPV2 downregulation. Data represented the mean ± SEM of a minimum of three independent experiments. Statistical significance ***: p-value < 0.0005.

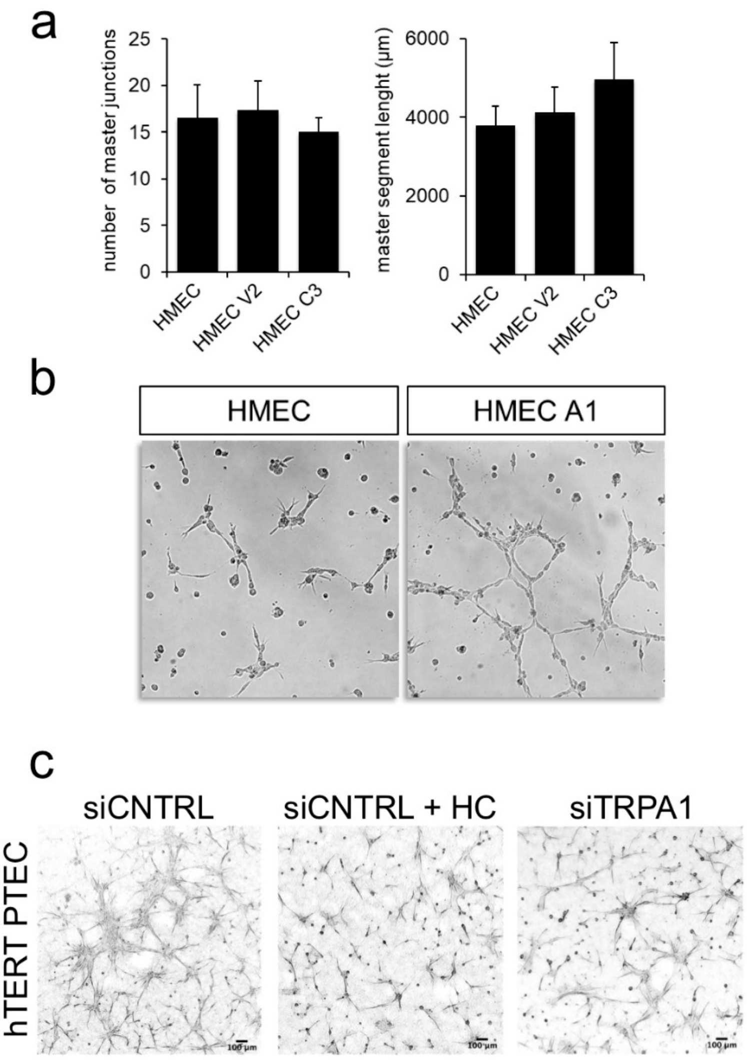

Figure S5. Validation of '*prostate-associated*' channel in tubulogenesis in vitro. (**a**) Among all the 'prostate-associated' channels, no effect on the capillary-like structure formation was observed for TRPV2- or TRPC3-overexpressing HMECs compared to control cells. Bar graphs show the quantification of total master segment length and the number of master junctions of control HMEC and TRPV2- or TRPC3-overexpressing HMEC. Three independent experiments were performed 24 h after transfection. (**b**) representative images of CNTRL or TRPA1-overexpressing HMEC 20 h after Matrigel seeding; (**c**) representative images of CNTRL or TRPA1-downregulating hTERT PTEC 20 h after Matrigel/collagenI substrate seeding.

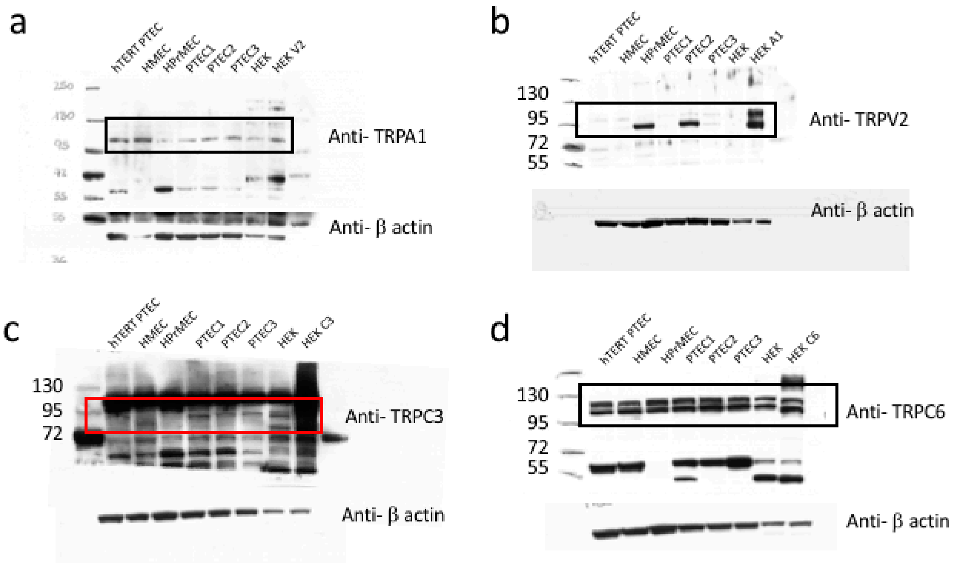

Figure S6. Whole scans of the immunoblots used in Figures 4 and 2S showing TRPA1 (**a**), TRPV2 (**b**), TRPC3 (**c**), and TRPC6 (**d**) expression in hTERT PTEC, HMEC, HPrMEC, PTEC1, 2 and 3, HEK, and overexpressing each channel. B-actin was used as a loading control.

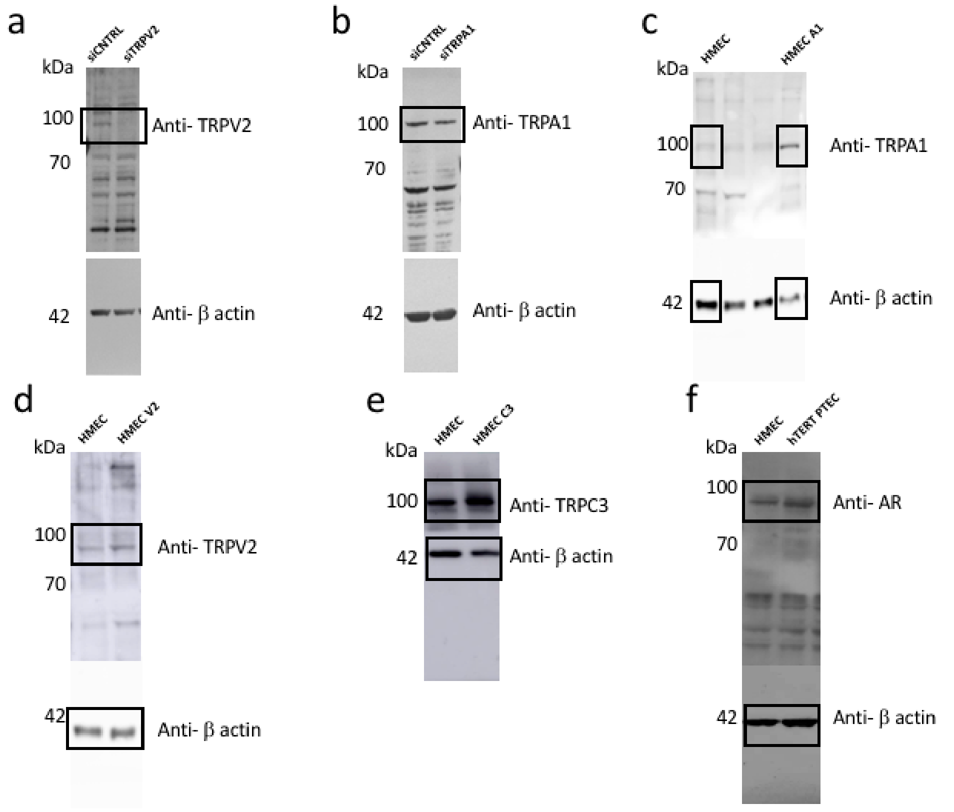

Figure S7. Whole scans of the immunoblots used in Figures 3, 5, S3 and S4 showing siRNA knochdown of TRPV2 (**a**), and TRPA1 (**b**); overexpression of TRPA1 (**c**), TRPV2 (**d**) and TRPC3 (**e**) in HMEC, as well as AR expression in HMEC and hTERT (**f**). B-actin was used as a loading control.

© 2019 by the authors. Licensee MDPI, Basel, Switzerland. This article is an open access article distributed under the terms and conditions of the Creative Commons Attribution (CC BY) license (http://creativecommons.org/licenses/by/4.0/).
